# Response in axillary lymph nodes to neoadjuvant chemotherapy for breast cancers: correlation with breast response, pathologic features, and accuracy of radioactive seed localization

**DOI:** 10.1007/s10549-023-06983-3

**Published:** 2023-06-08

**Authors:** Beth Z. Clark, Ronald R. Johnson, Wendie A. Berg, Priscilla McAuliffe, Rohit Bhargava

**Affiliations:** 1 Department of Pathology, UPMC Magee-Womens Hospital, 300 Halket St., Pittsburgh, PA 15213 USA; 2 Department of Surgery, UPMC Magee-Womens Hospital, 300 Halket St., Pittsburgh, PA 15213 USA; 3 Department of Radiology, University of Pittsburgh School of Medicine, UPMC Magee-Womens Hospital, 300 Halket St., Pittsburgh, PA 15213 USA

**Keywords:** Breast carcinoma, Neoadjuvant chemotherapy, Lymph node response

## Abstract

**Objectives:**

This study examined the accuracy of radioactive seed localization (RSL) of lymph nodes (LNs) following neoadjuvant chemotherapy (NAC) for invasive breast carcinoma, recorded pathologic features of LNs following NAC, evaluated concordance of response between breast and LNs, and identified clinicopathologic factors associated with higher risk of residual lymph node involvement.

**Methods:**

Clinical records, imaging, and pathology reports and slides were retrospectively reviewed for 174 breast cancer patients who received NAC. Chi-square and Fisher’s exact tests were used to compare differences in risk of residual lymph node disease.

**Results:**

Retrieval of biopsied pre-therapy positive LN was confirmed in 86/93 (88%) cases overall, and in 75/77 (97%) of cases utilizing RSL. Biopsy clip site was the best pathologic feature to confirm retrieval of a biopsied lymph node. Pre-therapy clinical N stage > 0, positive pre-therapy lymph node biopsy, estrogen and progesterone receptor positivity, Ki67 < 50%, HR + /HER2− tumors, and residual breast disease had higher likelihood of residual lymph node disease after NAC (*p* < 0.001).

**Conclusions:**

RSL-guided LN excision improves retrieval of previously biopsied LNs following NAC. The pathologist can use histologic features to confirm retrieval of targeted LNs, and tumor characteristics can be used to predict a higher risk of residual LN involvement.

## Introduction

Post-therapy lymph node (LN) status is an important prognostic factor in patients who receive neoadjuvant chemotherapy (NAC) for breast cancer, as residual tumor in lymph nodes is associated with reduced survival and increased risk of locoregional recurrence [1–7]. Residual lymph node involvement is also an important component of the Residual Cancer Burden (RCB) calculation, which is widely used to assess prognosis after NAC [8]. Therefore, accurate assessment of post-therapy lymph node status is important for prognosis and treatment decisions. The morbidity of axillary lymph node dissection (ALND) has encouraged the development of techniques to minimize axillary surgery in breast cancer after NAC while ensuring a clear picture of response to treatment. Targeted lymph node excision coupled with sentinel lymph node biopsy (SLNB) utilizes placement of a biopsy marker clip into the axillary lymph node (ALN) prior to NAC, radioactive seed-localization (RSL) of lymph nodes prior to surgery, and specimen radiographs to document retrieval of the clipped lymph node along with the radioactive seed. This approach allows pathologic evaluation of previously involved lymph nodes and has been shown to decrease the false negative rate of SLNB alone [9, 10]. As part of the multidisciplinary care team, along with surgeons and radiologists, surgical pathologists play an important role in documenting the presence of residual lymph node involvement, and can also use histologic features to confirm whether a biopsied lymph node has been retrieved.

Given the clinical importance of residual ALN involvement after NAC, the aims of this study were to evaluate accuracy of RSL of ALNs at our institution, to compare the response to NAC in the breast and in sampled ALNs, and to identify pathologic factors associated with a higher risk of residual lymph node disease.

## Methods

Following approval by the Institutional Review Board, a Copath Natural Language search of the institutional pathology database was used to identify post-NAC surgical specimens from 2017 to 2019 by searching for the synoptic report generated for surgical pathology specimens obtained after NAC. Initial Copath search for neoadjuvant synoptic was performed for dates encompassing 1/1/2018–03/31/2019. To increase the yield of cases with known pre-therapy LN involvement, an additional search for cases with neoadjuvant synoptic and positive pre-therapy LN core biopsy was performed to for dates encompassing 01/01/2017–12/31/2017. Cases of de novo metastatic disease and neoadjuvant hormonal therapy were excluded.

Our usual practice for evaluation of ALNs includes diagnostic axillary handheld ultrasound at the time of ultrasound evaluation of a suspicious breast finding. Ultrasound-guided 14-g core biopsy is performed at the request of treating physicians and we place a HydroMARK clip (Leica Biosystems, Buffalo Grove, IL, containing hydrogel) into the biopsied node. A post-procedure mammogram is performed. Within five days prior to surgery, our usual practice is to perform ultrasound-guided ^125^I-radioactive seed localization (Best Medical International, Springfield, VA) of the biopsied node. The clip is typically visible even when the node has normalized morphologically. A post-procedure mammogram is performed. During surgery, a hand-held gamma probe is used to identify the node containing the seed, with the goal of excising that node and two others. Magnification digital specimen radiography is usually performed, reviewed by the radiologist to confirm retrieval of the clip, seed, and node(s), and results telephoned to the operating room.

At our institution, pathologic evaluation consists of entirely submitting the tumor bed in the breast when gross residual tumor is not identified. Sentinel lymph nodes are sectioned at 2-mm intervals and entirely submitted for histologic examination. Specimen radiographs and Faxitron imaging (Hologic, Inc. Tuscon, AZ) are available to prosectors during specimen processing. Immunohistochemistry, typically cytokeratin AE1/AE3 (Leica Microsystems, Deerfield, IL), is utilized to identify residual tumor cells in difficult cases.

Pathology reports were reviewed to record type of breast and ALN surgical procedures, including utilization of radioactive seed localization (RSL), along with tumor type, tumor grade, biomarker status, response to therapy, and residual cancer burden (RCB). Clinical data were recorded from the electronic medical record, including age, clinical stage, neoadjuvant regimen, imaging response to NAC, recurrence, and status at last follow-up.

Confirmation of biopsied LN retrieval was assessed by documentation of the presence of a biopsy clip either on specimen radiograph performed by radiology, biopsy clip identification during gross examination of the lymph nodes, and/or pathologic features of biopsy site changes. Pathology slides from cases with a positive pre-therapy LN biopsy were reviewed by a breast pathologist (BC).

For the purposes of this study, residual lymph node involvement was defined as the presence of any documented residual tumor cells, including isolated tumor cells (itcs), as some studies have demonstrated that residual itcs have prognostic significance [11]. Histologic features associated with prior LN biopsy, including a biopsy tract, defined as a focus of linear fibrosis at the periphery of the LN or in the surrounding adipose tissue, and biopsy clip site, defined as a space in the tissue lined by palisading histiocytes or as eosinophilic or basophilic biodegradable carrier material (BCM) surrounded by histiocytes and fibrosis, were recorded. The location of the biopsy clip site (inside or outside the LN) was also recorded. Imaging reports and electronic medical records were reviewed to investigate cases in which retrieval of biopsied LN could not be confirmed pathologically, and imaging was reviewed by a breast imaging radiologist (WB).

The association between various clinical-pathological variables and presence of residual carcinoma in the lymph nodes was analyzed. Univariable analysis was performed using χ2 and Fisher’s exact tests to compare the differences in percentages between groups. A *p*-value < 0.05 was considered significant. Risk ratios were calculated for each of the variables for risk of residual disease in the lymph node. The variables showing statistically significant differences were included in multinomial logistic regression analysis. Statistical analysis was performed using IBM SPSS Statistics for Windows, Version 27.0. Armonk, NY: IBM Corp.

## Results

Copath Natural Language Search yielded 181 cases for possible inclusion in the study. One case was excluded because it represented an axillary recurrence of carcinoma. One case was excluded because the patient received neoadjuvant hormonal therapy along with NAC. One case was excluded because a pre-therapy excisional LN biopsy was performed, and four cases were excluded because the patient presented with de novo metastatic breast carcinoma, yielding 174 cases: 93 cases had biopsy-documented metastatic carcinoma in an ALN, 35 cases had a negative pre-NAC biopsy of an ALN, and in 46 cases, no pre-therapy ALN biopsy was performed. Clinical data are reported in Table 1, and pathology data are reported in Table 2. Pathology slides were available for review in 85/93 cases (91%) with positive pre-therapy LN biopsy. For the remaining eight cases, pathology report findings were included in the analysis where appropriate. Pathology slides for 6 cases with negative pre-therapy LN core biopsy but positive LNs after NAC were also reviewed. For simplicity, “biopsied lymph node” refers to an LN that was sampled by percutaneous core biopsy prior to NAC.

**Table 1:** Clinical characteristics for 174 cases of breast carcinoma status post neoadjuvant chemotherapy

		Pre-therapy lymph node biopsy positive (n = 93)	Pre-therapy lymph node biopsy negative (n = 35)	No pre-therapy lymph node biopsy (n = 46)	Total
Median Age (range)		54 (24–78)	43 (28–71)	53 (30–82)	51 (24–82)
Clinical Tumor stage (cT)	1	11 (12%)	6 (17%)	7 (15%)	24 (14%)
	2	58 (62%)	27 (77%)	32 (70%)	117 (67%)
	3	17 (18%)	2 (6%)	4 (9%)	23 (13%)
	4	7 (8%)	0 (0%)	2 (4%)	9 (5%)
	unknown	0 (0%)	0 (0%)	1 (2%)	1 (1%)
Clinical Node stage (cN)	0	0 (0%)	30 (86%)	39 (85%)	69 (40%)
	1	77 (83%)	3 (8%)	4 (9%)	84 (48%)
	2	11 (12%)	0 (0%)	2 (4%)	13 (7%)
	3	5 (5%)	0 (0%)	0 (0%)	5 (3%)
	unknown	0 (0%)	2 (6%)	1 (2%)	2 (2%)
Breast surgery	Breast-conserving surgery	44 (47%)	6 (17%)	20 (43%)	70 (40%)
	Total mastectomy	47 (51%)	17 (49%)	16 (35%)	80 (46%)
	Skin-sparing mastectomy	1 (1%)	4 (11%)	7 (15%)	12 (7%)
	Nipple-sparing mastectomy	1 (1%)	8 (23%)	3 (7%)	12 (7%)
Chemotherapy completed		75 (81%)	29 (83%)	34 (74%)	138 (79%)
Recurrence	Locoregional	1 (1%)	0 (0%)	4 (8%)	5 (3%)
	Distant	11 (12%)	4 (11%)	1 (2%)	16 (9%)
	Locoregional and distant	0 (0%)	0 (0%)	1 (2%)	1 (1%)
Status at last follow-up	NED	79 (85%)	30 (85%)	38 (83%)	147 (84%)
	AWD	7 (8%)	2 (6%)	5 (11%)	14 (8%)
	DOD	5 (5%)	2 (6%)	1 (2%)	8 (5%)
	DOC	2 (2%)	1 (3%)	2 (4%)	5 (3%)

*NED* no evidence of disease, *AWD* alive with disease, *DOD* died of disease, *DOC* died of other causes

**Table 2:** Pathologic characteristics for 174 cases of breast carcinoma status post neoadjuvant chemotherapy

		Pre-therapy lymph node biopsy positive (n = 93)	Pre-therapy lymph node biopsy negative (n = 35)	No pre-therapy lymph node biopsy (n = 46)	Total
Histologic tumor type	Ductal, NST	82 (89%)	30 (86%)	38 (83%)	150 (86%)
	Lobular	5 (5%)	1 (3%)	2 (4%)	8 (5%)
	Mixed Ductal/Lobular	2 (2%)	1 (3%)	2 (4%)	5 (3%)
	Metaplastic	1 (1%)	2 (5%)	3 (7%)	6 (3%)
	Special types (mucinous, neuroendocrine, or micropapillary)	3 (3%)	1 (3%)	1 (2%)	5 (3%)
Pre-therapy Estrogen Receptor (ER) Status	Positive	59 (63%)	20 (57%)	22 (48%)	101 (58%)
	Negative	34 (37%)	15 (43%)	24 (52%)	73 (42%)
Pre-therapy Progesterone Receptor (PR) Status	Positive	45 (48%)	24 (69%)	16 (35%)	85 (49%)
	Negative	48 (52%)	11 (31%)	30 (65%)	89 (51%)
Pre-therapy HER2 Status	Positive	28 (30%)	12 (34%)	14 (30%)	54 (31%)
	Negative	65 (70%)^a^	23 (66%)	32 (70%)^b^	120 (69%)
Pre-therapy Tumor Phenotype	HR + /HER2−	39 (42%)	7 (20%)	19 (41%)	65 (37%)
	HR + /HER2 +	19 (20%)	8 (23%)	5 (11%)	32 (18%)
	HR-/HER2+	9 (10%)	4 (11%)	3 (7%)	16 (9%)
	HR-/HER2−	26 (28%)	16 (46%)	19 (41%)	61 (36%)
Post-therapy Nottingham Grade	Grade 1	3 (3%)	1 (3%)	4 (10%)	8 (5%)
	Grade 2	36 (39%)	8 (23%)	14 (30%)	58 (33%)
	Grade 3	23 (25%)	7 (20%)	14 (30%)	44 (25%)
	No residual	28 (30%)	19 (54%)	12 (26%)	59 (34%)
	Unknown	3 (3%)	0 (%)	2 (4%)	5 (3%)
Post-therapy pathologic tumor stage (ypT)	ypT0	24 (26%)	17 (48%)	10 (21%)	51 (29%)
	ypTis	3 (3%)	2 (6%)	2 (4%)	7 (4%)
	ypT1mi	3 (3%)	2 (6%)	3 (7%)	8 (5%)
	ypT1a	11 (12%)	2 (6%)	9 (20%)	22 (13%)
	ypT1b	6 (6%)	2 (6%)	3 (7%)	11 (6%)
	ypT1c	10 (11%)	2 (6%)	8 (17%)	20 (11%)
	ypT2	23 (25%)	6 (16%)	9 (20%)	38 (22%)
	ypT3	12 (13%)	2 (6%)	2 (4%)	16 (9%)
	ypT4	1 (1%)	0 (0%)	0 (0%)	1 (1%)
Post-therapy pathologic nodal stage (ypN)	ypN0	37 (40%)	28 (80%)	40 (87%)	105 (61%)
	ypN0i +	2 (2%)	2 (6%)	0 (0%)	4 (2%)
	ypN1mic	7 (8%)	2 (6%)	0 (0%)	9 (5%)
	ypN1	27 (29%)	3 (8%)	3 (7%)	33 (19%)
	ypN2	14 (15%)	0 (0%)	2 (4%)	16 (9%)
	ypN3	6 (6%)	0 (0%)	1 (2%)	7 (4%)
Residual Cancer Burden (RCB) Category	pCR	25 (27%)	19 (54%)	12 (26%)	56 (32%)
	RCB-I	10 (11%)	2 (6%)	8 (17%)	20 (12%)
	RCB-II	26 (28%)	13 (37%)	24 (53%)	63 (36%)
	RCB-III	32 (34%)	1 (3%)	2 (4%)	35 (20%)

*HR* hormone receptor

^a^Using ASCO/CAP 2018 focused update criteria. Nine cases were previously categorized as equivocal

^b^Using ASCO/CAP 2018 focused update criteria. Four cases were previously categorized as equivocal. Biomarker status

### Pathologic features of previously biopsied lymph nodes following neoadjuvant chemotherapy

Histologic review of the breast and LNs in cases with known positive lymph nodes demonstrated that therapy-related changes were more frequently observed in the breast (82/85, 96% cases) than the sampled LNs (67/85, 79%). The most frequently observed change in the breast included loose fibroelastosis (76/85, 89%), followed by lymphocyte aggregates in 31/85 (36%), dense homogeneous fibrosis in 30/85 (35%), hemosiderin-laden macrophages in 23/85 (27%), and macrophage aggregates in 21/85 (25%).

**Fig. 1:**
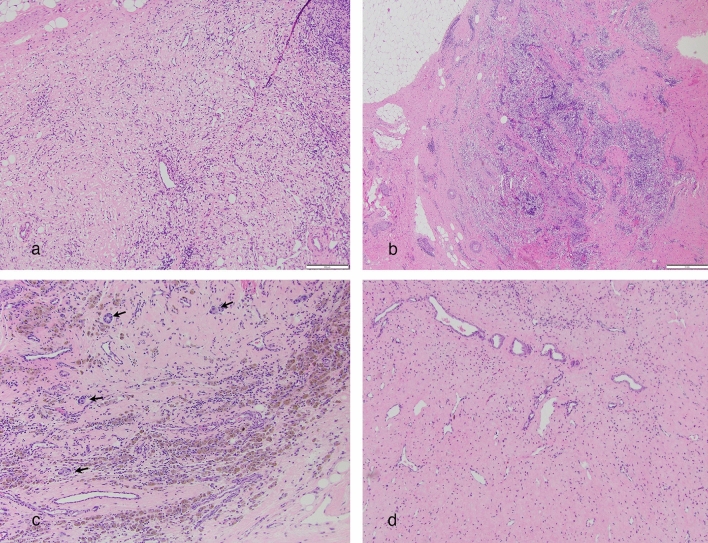
Therapy-related changes within a lymph node can have a variety of appearances. Loose fibroelastosis with delicate vasculature (**a**); Lymph node with numerous hemosiderin-laden macrophages and no residual carcinoma (**b**); abundant hemosiderin-laden macrophages with scattered minute foci of residual carcinoma (arrows) (**c**); dense homogeneous fibrosis of lymph node with no residual carcinoma (**d**)

Within the sampled LNs, loose fibroelastosis was identified in 47/85 (55%), dense homogeneous fibrosis in 40/85 (47%), macrophage aggregates in 21/85 (25%), and hemosiderin-laden macrophages in 13/85 (15%). (Fig. 1).

**Fig. 2:**
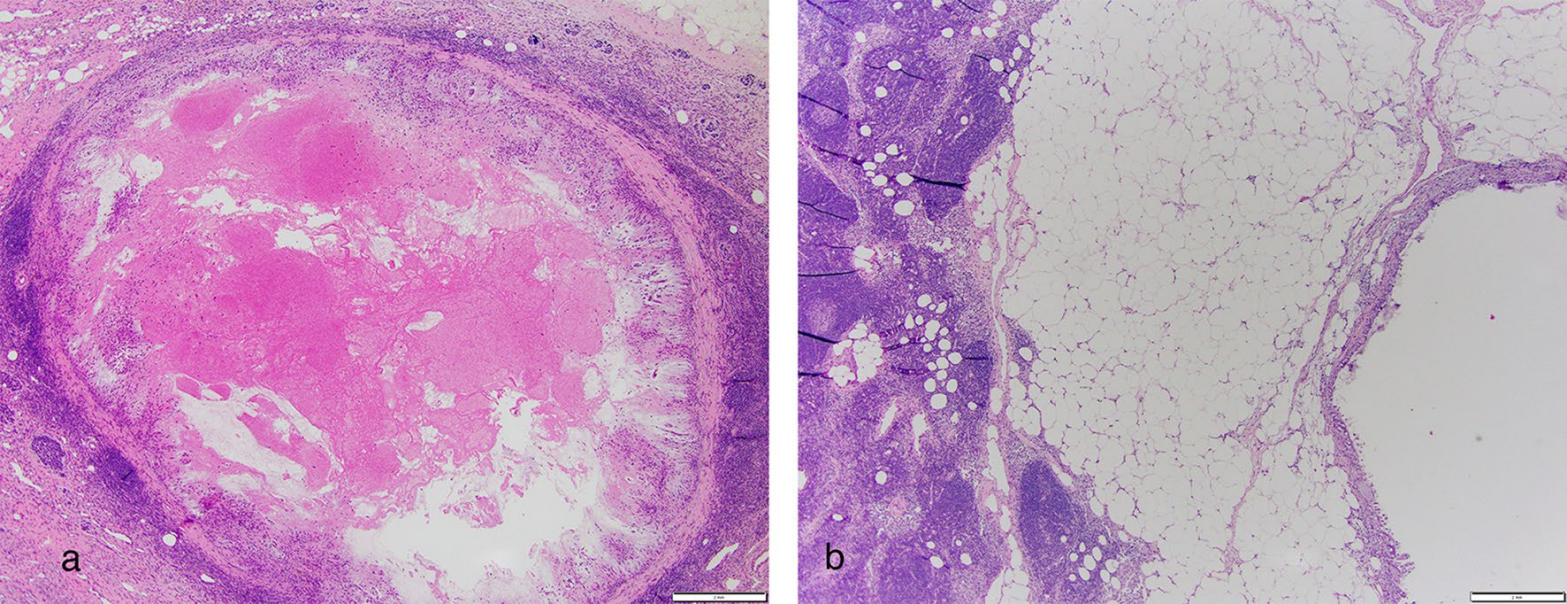
Biopsy clip site may be identified inside (**a**) or outside (**b**) the biopsied lymph node

**Fig. 3:**
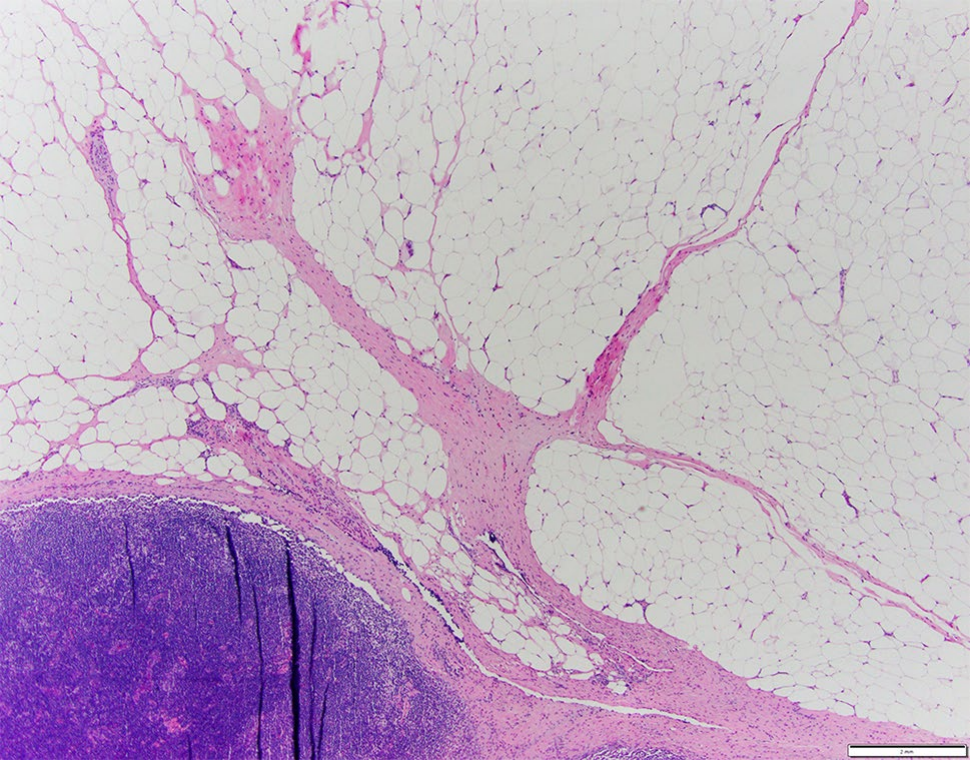
Linear fibrosis in perinodal adipose tissue, compatible with biopsy tract

**Fig. 4:**
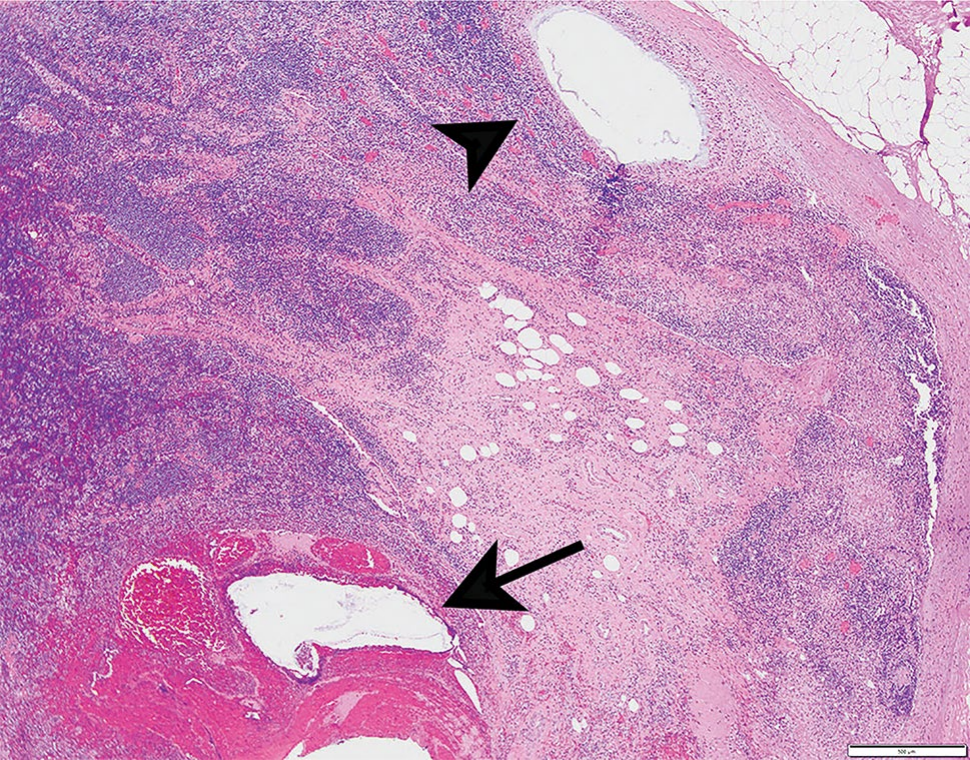
Radioactive seed site (arrow) and biopsy clip site (arrowhead) in previously biopsied lymph node

The biopsy clip site was identified in the LN in 69/85 (81%) cases reviewed and was the most frequently identified biopsy site change. The biopsy clip site was present within the LN in 50 cases, adjacent to the LN in 12 cases, and seen both inside and adjacent to the LN in 7 cases (Fig. 2). A biopsy tract was identified in 37/85 (43%) cases (Fig. 3). The radioactive seed site was characterized by acute hemorrhage with associated acute inflammation and could be distinguished from the biopsy clip site histologically (Fig. 4).

Of the 93 cases with a positive pre-NAC biopsied LN, 38 showed no residual carcinoma in the LNs. Of 35 cases with slides available for review, therapy-related changes were identified in the biopsied LN in 25, no therapy-related changes were identified in six, and findings were indeterminate in four. The six cases without therapy-related changes consisted of two HR-/HER2−, two HR-/HER2+ , and two HR + /HER2+ breast carcinomas. In an additional four cases, findings were indeterminate, with focal fibrosis adjacent to the biopsy site, but no definite tumor-bed like fibrosis was identified.

### Accuracy of radioactive seed localization in retrieval of previously biopsied axillary lymph nodes

**Fig. 5:**
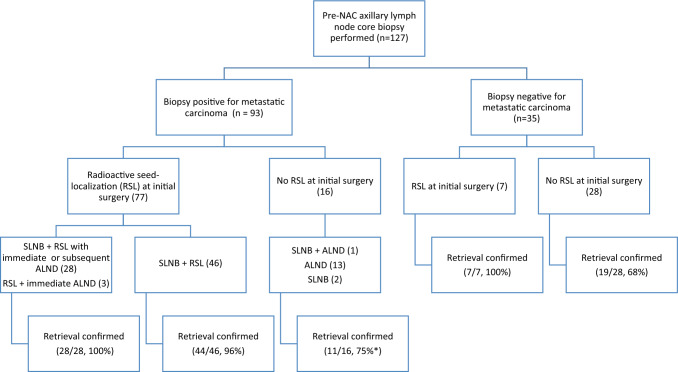
Flowchart of cases with pre-therapy lymph node biopsy and confirmation of lymph node retrieval during initial surgical procedure. *Note for positive pre-therapy biopsy but no RSL performed: In 3 cases, no biopsy clip was placed; in one case, initial biopsy was likely of a soft tissue mass rather than a lymph node, and in one case, biopsy clip was placed and no evidence of biopsy clip or biopsy site changes was identified in ALND (Faxitron imaging performed of axillary tail)

**Fig. 6:**
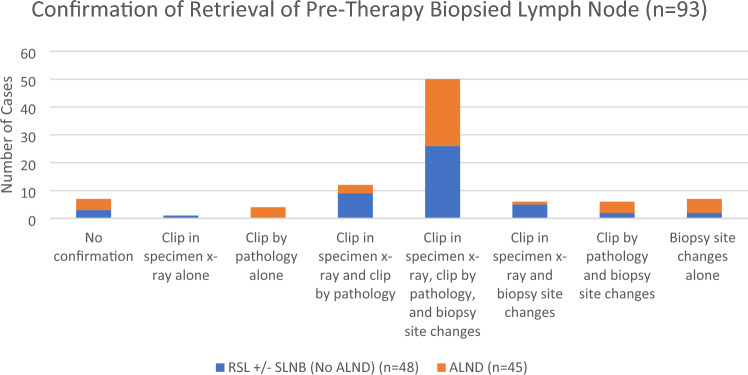
Confirmation of retrieval of previously biopsied lymph nodes can be achieved through documentation of biopsy clip on specimen x-ray or gross examination in pathology or through demonstration of biopsy site changes on routine hematoxylin and eosin (H&E)-stained slides. The RSL (radioactive seed localization) ± SLNB (sentinel lymph node biopsy) category refers to cases without formal axillary lymph node dissection (ALND), and the ALND category refers to cases in which formal ALND was ultimately performed, either as the initial surgical procedure or following RSL ± SLND. Note: In the RSL ± SLNB category, pathology slides were not available for review in four cases, and 2 cases had no specimen x-ray. In the ALND category, pathology slides were not available for review in one case, and specimen x-ray was not performed in fourteen cases. In two cases in the “No confirmation” category, biopsy site changes were identified in adipose tissue not associated with an adjacent lymph node

Figure 5 details LN surgeries, utilization of RSL, and confirmation of biopsied LN retrieval for patients with positive and negative pre-NAC LN biopsies. Figure 6 details the presence of a biopsy clip in specimen radiograph, clip retrieved by pathology, and presence of biopsy site changes in patients with positive pre-therapy biopsy.

Of the 93 cases with positive pre-NAC biopsy, regardless of the LN surgery performed, retrieval of the biopsied LN was confirmed by pathology in the initial surgical procedure in 84/93 cases (90%) through a combination of clip retrieval and/or the presence of biopsy site changes. Additional RSL procedures were performed in three cases, and the biopsied LN was identified in 2 cases; therefore, retrieval was ultimately confirmed in 86/93 cases (92%).

For the 77 cases in which RSL of the biopsied lymph node was performed in the initial surgical procedure, specimen radiography was performed by breast imaging in 75. Retrieval of the biopsied lymph node was confirmed by pathology in 73 cases (95%) and not confirmed in four. In one of these four cases, the biopsy clip was identified in specimen radiograph, suggesting that the clip and biopsy site may have been lost during frozen section microtomy, and in another case, a second RSL procedure was performed, and the biopsied LN was retrieved; therefore, retrieval was ultimately confirmed in 75/77 (97%) cases. The presence of a biopsy clip in a lymph node specimen was documented in the pathology report in 70/91 (77%) cases in which a clip was placed. In 12 of the 21 cases without a clip documented at pathology, a biopsy clip was documented on specimen radiograph, suggesting that the biopsy clip was dislodged during sectioning (11 cases), or lost in frozen section microtomy (one case, referenced above).

Of 86 cases with confirmed removal of the biopsied known malignant lymph node, 47 (55%) had residual involvement of the biopsied LN, one showed involvement of a different LN, and 38 (44%) had no residual LN involvement. Among the 47 cases with residual involvement of the biopsied LN, 31 (66%) had additional positive LNs, while in 16 cases (34%), the biopsied LN was the only sampled node with residual disease.

Of 35 cases with a negative lymph node biopsy prior to NAC, retrieval of the biopsied LN was confirmed through presence of biopsy clip and/or biopsy site changes in 26/35 (74%) cases overall, and 7/7 cases (100%) in which RSL was utilized. Positive LNs were identified in 6/35 cases (17%): 2 itcs, 2 N1mic, 2 N1a. Itcs were identified in the biopsied LN in one case, a LN other than the biopsied LN in four cases, and one case was indeterminate. Four cases with previously unknown nodal disease had HR + /HER2− primaries and two were HR + /HER2 + .

### Correlation of neoadjuvant chemotherapy response in breast and sampled axillary lymph nodes

Pathologic complete response (pCR), defined as absence of invasive carcinoma in breast and LNs, was reported in 56/174 (32%) cases. Correlation between the presence of residual invasive carcinoma in the breast and lymph nodes is reported in Table 3. Among 58 cases with no residual invasive carcinoma in the breast (ypT0 and ypTis), itcs were identified in lymph nodes in 2/58 cases (3%) with no micro- or macrometastases identified. Both cases with itcs had positive pre-NAC LN biopsy, and itcs were identified in the biopsied LN. Completion ALND was not performed in either case. Complete response in the ALNs, but not the breast, was identified in 12 cases with positive pre-NAC lymph node biopsy (10 cases ypT1, 2 cases ypT2).

**Table 3:** Correlation of residual carcinoma in breast and sampled axillary lymph nodes (ALNs)

	Pre-therapy ALN biopsy positive (n = 93)	Pre-therapy ALN biopsy negative (n = 35)	No pre-therapy ALN biopsy (n = 46)	Total
No residual invasive disease in breast or ALNs (pCR)	25 (27%)	19 (54%)	12 (26%)	56 (32%)
Residual disease in both breast and ALNs	54 (58%)	7 (20%)	6 (13%)	67 (39%)
Residual Disease in Breast Only	12 (13%)	9 (26%)	28 (61%)	49 (28%)
Residual Disease in ALNs Only	2 (2%)^a^	0 (0%)	0 (0%)	2 (1%)

^a^Isolated tumor cells (itcs) only

Multiple factors were associated with a higher likelihood of residual positive ALNs after NAC (Table 4). Clinical N stage > 0, positive pre-therapy LN biopsy, ER positivity, PR positivity, Ki67 proliferative index < 50%, and patients with HR + /HER2 tumors predicted higher risk of residual lymph node disease. Residual disease in the breast (ypT ≥ 1) was also associated with a higher risk of residual LN involvement. Variables significant on univariable analysis were included for multinomial logistic regression analysis (ER status, PR status, Ki-67 proliferation index, cN stage, pre-therapy LN biopsy status, IHC tumor class, ypT stage) (Table 5). Clinical N stage and ypT stage variables were statistically significant, indicating that patients with negative clinical nodal status prior to NAC and absence of residual disease in the breast post-therapy are less likely to have residual lymph node disease.

**Table 4:** Clinicopathologic variables and association with residual lymph node involvement after neoadjuvant chemotherapy

Variables	Residual disease in lymph node	No residual disease in lymph node	*p*-value	Risk ratio (95% CI)
Histology				
IDC, NST (n = 150)	56/150 (37%)	94/150 (63%)	0.265	0.747
Other (n = 24)	12/24 (50%)	12/24 (50%)		(0.476–1.172)
ER				
Negative (n = 78)	15/78 (19%)	63/78 (81%)	< 0.001*	0.348
Positive (n = 96)	53/96 (55%)	43/96 (45%)		(0.214–0.568)
PR				
Negative (n = 102)	26/102 (26%)	76/102 (76%)	< 0.001*	0.437
Positive (n = 72)	42/72 (58%)	30/72 (42%)		(0.297–0.642)
HER2				
Negative (n = 120)	52/120 (43%)	68/120 (57%)	0.096	1.463
Positive (n = 54)	16/54 (30%)	38/54 (70%)		(0.924–2.315)
Ki-67 proliferation index				
50% or less (n = 61)	36/61 (59%)	25/61 (41%)	< 0.001*	1.973
> 50% (n = 107)	32/107 (30%)	75/107 (70%)		(1.380–2.822)
Clinical T-stage				
cT1 (n = 24)	8/24 (33%)	16/24 (67%)	0.654	0.828
cT2/3/4 (n = 149)	60/149 (40%)	89/149 (60%)		(0.455–1.506)
Clinical N-stage				
cN0 (n = 69)	7/69 (10%)	62/69 (90%)	< 0.001*	0.170
cN1/2/3 (n = 102)	61/102 (60%)	41/102 (40%)		(0.083–0.349)
Pre-therapy LN biopsy				
Negative/NP (n = 81)	12/81 (15%)	69/81 (85%)	< 0.001*	0.246
Positive (n = 93)	56/93 (60%)	37/93 (40%)		(0.142–0.425)
IHC tumor class				
HR + /HER2-neg (n = 61)	39/61 (64%)	22/61 (36%)	< 0.001*	2.491
Other (n = 113)	29/113 (26%)	84/113 (74%)		(1.728–3.592)
Post-therapy pT-stage				
ypT0 (n = 58)	2/58 (3%)	56/58 (97%)	< 0.001*	0.061 (0.015–0.239)
ypT1/2/3/4 (n = 116)	66/116 (57%)	50/116 (43%)

**Table 5:** Results of multinomial logistic regression analysis – clinicopathologic variables associated with higher risk of residual lymph node involvement after neoadjuvant chemotherapy

Variables	Regression Coefficient	*p*-value	Odds ratio (95% CI)
ER status (negative versus positive)	− 1.095	0.198	0.335 (0.063–1.770)
PR status (negative versus positive)	0.794	0.305	2.211 (0.486–10.064)
Ki-67 proliferation index (≤ 50% versus > 50%)	0.673	0.264	1.960 (0.601–6.385)
cN stage (cN0 versus cN1/2/3)	− 2.587	0.005*	0.075 (0.012–0.461)
Pre-Rx LN biopsy (Negative/NP versus positive)	− 0.905	0.260	0.404 (0.084–1.957)
IHC tumor class (ER + /HER2-neg versus others)	1.113	0.092	3.044 (0.833–11.122)
ypT stage (ypT0 versus ypT1/2/3/4)	− 4.019	< 0.001*	0.018 (0.003–0.096)

*ER* estrogen receptor, *PR* progesterone receptor, *cN* clinical nodal stage, *LN* lymph node, *NP* not performed, *IHC* immunohistochemical, *ypT* post-therapy tumor stage, *CI* confidence interval

## Discussion

This study has shown that RSL-guided LN excision improves retrieval of previously biopsied LNs following NAC, and a multidisciplinary approach utilizing specimen radiography and documentation of biopsy clip and histologic features by pathology are complementary. Tumor characteristics can be used to predict a higher risk of residual LN involvement, allowing for individualized treatment.

In the setting of NAC, given the possibility of eradication of LN disease, particularly in certain tumor phenotypes, techniques to avoid ALND have become increasingly important. A National Cancer Database (NCDB) study of practice patterns of axillary management showed a decrease in the rate of ALND in patients who are clinically node-positive prior to NAC in both community and academic settings [12]. Commonly utilized alternatives to ALND include SLNB alone and SLNB plus targeted excision of previously biopsied LNs using RSL.

The false negative rate for SLNB following NAC has been evaluated in several large studies. In an analysis of 218 patients who underwent SLNB in addition to ALND in the National Surgical Adjuvant Breast and Bowel Project (NSABP) Protocol B-27, the false negative rate of SLNB was 10.7% [13]. In the SENTINA trial, the false negative rate (FNR) of SLNB for clinically node-positive patients who were clinically node-negative following NACT was 14.2% overall, but consistently less than 10% when three or more lymph nodes were retrieved [14]. The GANEA 2 trial reported an overall FNR of 11.9% for SLNB in 244 patients with documented lymph node involvement prior to NACT, and 7.8% when two or more SLNs were identified [15]. In ACOSOG Z1071, in which all patients had histologically confirmed lymph node involvement by core biopsy or fine needle aspiration biopsy, the false negative rate for SLNB following NACT was 12.6% with at least two SLNs removed, but significantly decreased with three or more SLNs were removed [16]. Later, Boughey, et al. published an analysis of the impact of clip placement on the false negative rate of SLNB [9]. Of 203 cN1 patients with a clip in the biopsied lymph node, the clip location was known in 141 cases. The clip was identified in an SLNB specimen in 107 (75.9%) of cases with false negative rate (FNR) of 6.8%, but in the 34 (24.1%) cases in which the clipped LN was in the ALND, the FNR of SLNB was 19%, suggesting that localization of the clipped lymph node can decrease the FNR while avoiding ALND in patients without residual lymph node disease. In a study of 208 clinically node-positive patients, Caudle, et al. showed decreased FNR from 10.1% with SLNB to 1.4% when SLNB was combined with retrieval of the clipped node using RSL [10]. The clipped node was not retrieved as a SLN in 23% of patients and in six patients, the SLNs were negative but the clipped node was positive.

In a nonoverlapping earlier study from our institution, Diego, et al. described successful RSL of clipped LNs in 29/30 (97%) patients who were clinically node-negative following completion of NACT [17]. Residual carcinoma was identified in the clipped LN in 11/30 (37%) patients and no patients had other pathologically positive LNs when the seed-localized lymph node was negative. In the current study, regardless of the LN surgery performed, the LN with known pre-NAC involvement was identified in a high percentage of cases (92%) and was increased with utilization of RSL (97%). The biopsy clip was not identified by pathology in a substantial number of cases, although biopsy site changes were present. This suggests that loss of the biopsy clip on lymph node sectioning is not uncommon, and that biopsy site changes and biopsy clip site are helpful adjuncts to the identification of a biopsied LN. As shown in Fig. 6, biopsy clip identification on specimen radiograph, biopsy clip retrieval in pathology, and histologic identification of biopsy site changes can be present in various combinations, and pathology and specimen radiography are complementary in confirming retrieval of biopsied LNs.

RSL was not frequently performed when the pre-NAC LN biopsy was negative, but retrieval was confirmed in 74% of cases in this study. In one case, the biopsied LN showed itcs. Based on the low false-negative rate of percutaneous pre-NAC LN biopsy in this analysis (1/35 cases, < 3%), RSL of LNs with a negative pre-therapy core biopsy is likely not necessary unless clinical factors suggest otherwise; but there is a risk of residual low-volume residual LN disease, mostly in other LNs.

This study also evaluated the concordance of response to NAC in the breast and the ALNs. In patients with no residual invasive carcinoma in the breast, regardless of pre-therapy biopsy status, lymph nodes were negative in all but two ypT0 cases, which showed only itcs. In 13% of cases with positive pre-NAC LN biopsy, residual disease was present in the breast only. In cases with positive pre-NAC lymph node biopsy but no residual carcinoma in the LNs, definite therapy-related changes were identified in most (71%) cases, with possible therapy-related changes identified in an additional four cases. In six cases, however, no obvious tumor bed was identified, suggesting that in a minority of cases, complete resolution of metastatic carcinoma may occur without histologic evidence of prior involvement. Therefore, pre-NAC LN status cannot be determined with certainty after NAC based on histologic features alone. Of cases with a negative pre-NAC LN biopsy or no pre-NAC LN biopsy, residual ALN involvement was only identified in cases with residual disease in the breast.

Residual LN involvement following NAC is an adverse prognostic marker, even in cases with low-volume residual disease, and its presence often guides adjuvant therapy recommendations, including systemic therapy and radiation therapy [11, 18]. In this study, factors associated with lower risk of residual LN involvement after NAC included negative ER and PR status, Ki-67 proliferation index > 50%, clinical tumor size $\le$ 2 cm, cN0 status, ypT0, and negative pre-NAC LN biopsy as compared to positive pre-NAC LN biopsy. Patients with HR + /HER2-negative tumors had ~ 2.5 times the risk of having residual LN disease compared to patients with other tumors (risk ratio of 2.49). Samiei, et al., previously reported a lower rate of axillary clearance (45%) in 245 cN1 patients with breast pCR, and only 9.4% ypN0 without a breast pCR. The odds of ypN0 following neoadjuvant therapy were lower in cases with cT3, cN1, and ER + /HER2- subtype [19]. In a study of 191 patients with locally advanced breast cancer and cytologically confirmed axillary LN metastasis, predictors of eradication of axillary disease included ER negative tumors, smaller primary tumors, and no residual disease in the breast [20]. These findings have implications for the selection of patients for neoadjuvant systemic therapy as well as consideration of omission of surgery when an exceptional response to NAC has been demonstrated. Keurer, et al., demonstrated that the breast and ALN status were concordant in 97.5% of cases in a feasibility trial of exceptional responders to neoadjuvant systemic therapy [21]. In a prospective study of HER2 positive and triple negative breast cancer patients, designed to identify exceptional responders for consideration of omission of axillary surgery following NAC, residual disease in the breast was associated with a relative risk of positive nodal metastasis of 7.4 (95% CI, 3.7–14.8; *p* < 0.001) [22].

Based on this retrospective study, RSL of biopsied LNs is feasible, accurate, and can help to avoid the morbidity of ALND. Special handling of these LNs is required to identify the biopsy clip and biopsy site changes, as they may be in the perinodal adipose tissue. Similar histologic features of therapy response are seen in the breast and lymph nodes, and biopsy clip site can be used to confirm the retrieval of biopsied LNs when the clip has migrated or been dislodged during sectioning. Our study substantiates other studies showing that pCR in the breast is a strong predictor of complete response in the LNs, and that clinicopathologic factors, such as tumor phenotype and clinical nodal stage, can help to predict the likelihood of eradication of nodal disease.

## Data Availability

The datasets used and/or analyzed during the current study are available from the corresponding author on reasonable request.
